# Lymphoscintigraphy Defines New Lymphatic Pathways from Cutaneous Melanoma Site: Clinical Implications and Surgical Management

**DOI:** 10.1155/2011/817043

**Published:** 2011-12-25

**Authors:** Ugo Marone, Luigi Aloj, Gianluca Di Monta, Corrado Caracò

**Affiliations:** ^1^Department of Surgery “Melanoma, Soft Tissues, Head and Neck, Skin Cancers”, National Cancer Institute of Naples, 80131 Naples, Italy; ^2^Department of Nuclear Medicine, National Cancer Institute of Naples, 80131 Naples, Italy

## Abstract

Sentinel lymph node biopsy is commonly applied as staging procedure of regional lymph nodes in patients with cutaneous melanoma. Dynamic lymphoscintigraphy defines the lymphatic pathways from a primary melanoma site and allows to identify the node receiving lymphatic drainage from the primary tumor, which is the sentinel lymph node. In rare cases, lymphoscintigraphy shows sites of lymphatic drainage in nonclassical basins never described in the past when lymphatic drainage was considered only according to the anatomical proximity of the tumor primary site. These peculiar sentinel nodes, so-called “uncommon/interval” nodes, must be surgically removed because they may contain micrometastatic disease and may be the only site of nodal involvement.

## 1. Introduction

Sentinel lymph node biopsy (SLNB) is commonly applied as staging procedure of regional lymph nodes in patients with cutaneous melanoma. Dynamic lymphoscintigraphy defines the lymphatic pathways from a primary melanoma site and allows to identify the node receiving lymphatic drainage from the primary tumor, which is the sentinel lymph node [1, 2]. The combined modality of the radioactive colloid, blue dye, and an intraoperative gamma probe allows the biopsy of the sentinel node (SN), in order to detect microscopic nodal disease, with accurate selection of patients for early therapeutic lymphadenectomy or adjuvant therapy. Although most melanomas drain to the predicted classical anatomical basins (cervical, axillary, and inguinal nodes), some patients drain to lymph nodes in unpredicted sites, so-called interval nodes, aberrant nodes, intercalated, or unexpected nodes. These “not classical” sentinel nodes are those lymph nodes lying along the course of a lymphatic collecting vessel between the primary tumor site and the draining basin or that lymph nodes located in a nodal basin outside the anatomical predicted nodal field [3]. These peculiar sentinel nodes may contain micrometastatic disease and may be the only site of nodal involvement [4].

## 2. Sentinel Node Procedure

At the National Cancer Institute of Naples, patients with a primary tumor thicker than 1 mm or at least Clark level IV-V, ulcerated or with ≥1 mitosis/mm^2^, without clinical evidence of nodal metastases, undergo SLNB. Prior to surgery, we perform clinical evaluation of all patients with liver ultrasound, chest X-ray, and lactate dehydrogenase to rule out the presence of distant metastases. Patients who had received a wide excision of the primary (more than 3 cm) or had undergone reconstruction with a cutaneous rotation flap are excluded, because the probable disruption of lymphatic drainage. Dynamic lymphoscintigraphy is performed 2–4 hours before surgery. A dose of 18 MBq ^99m^Technetium-labelled colloidal albumin (nanocoll) colloid is injected intradermally around the tumor. Dynamic and planar images from different points of view are obtained, and the sentinel node “hot spot” is marked on the skin.

About 20 minutes before the surgical procedure, 1.0 mL of Patent Blue dye is injected intradermally around the primary scar. A hand-held gamma probe guides the identification of sentinel nodes, with correlation of radioactivity in vivo, ex vivo and in the operative field. Blue lymph nodes are excised as well any radioactive nodes that exhibit high level of radioactivity in the operative field. Serial sections of SN are analyzed by standard stain with hematoxylin and eosin (H&E) and immunohystochemical (IHC) staining with S-100 and HMB-45 antibodies. Patients with tumor- positive SN undergo complete lymph node dissection of the involved basin. Stage I-II melanoma patients were followed every three months for the first two years and every six months thereafter, with clinical evaluation, liver and lymph nodes ultrasound, serum biochemistry, and chest X-ray every six months. Computed tomography (CT) and fluorine 18 fluorodeoxyglucose (FDG) positron emission tomography (PET) and bone scans are performed only in cases of clinical suspicion of distant extension or recidiva. The follow up for stage III patients is similar to stage I-II patients, but a total body CT or FDG PET scan is performed every year [5].

**Figure 1:**
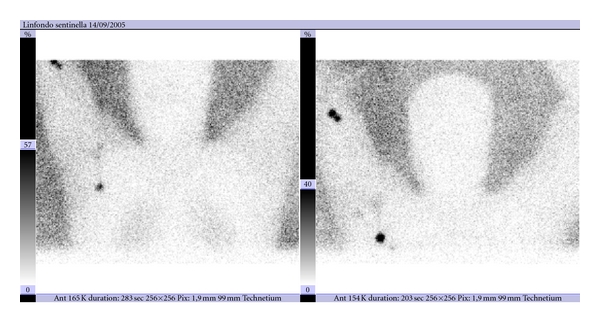
Lymphoscintigram of a patient with a primary melanoma of the forearm, with intense uptake of radioactive colloid in an epitrochlear node and uptake of the tracer in axillary region.

**Figure 2:**
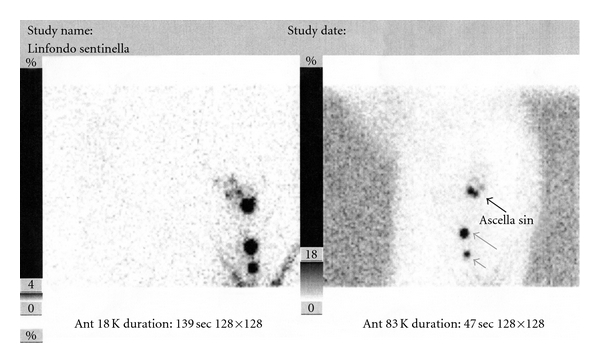
Lymphoscintigram of a patient with a primary melanoma in the left flank, demonstrating axillary nodal drainage, and an interval sentinel node at level of the chest wall.

The inguinal nodes are considered the classical nodal basin in the lower extremities, the axillary nodes are typical basin for the upper extremities. Drainage is expected to occur to the nearest axillary or inguinal basin in patients with primary tumor sites in the torso. If a melanoma is located within 2.5 cm of the midline, the drainage can occur to either side or both sides and is not considered discordant. Similarly, for melanomas located within 2.5 cm of the Sappey line, drainage can occur to the ipsilateral groin, axilla, or both [6]. For patients with head and neck primary melanoma all classical five levels in the neck are considered typical. Parotid, occipital and retroauricolar nodes are considered as possible standard sites of SN in head and neck melanomas [7]. All other “non classical” drainage sites can be classified according to the following terms: “uncommon” and “interval” sentinel node. An “uncommon” (Figure 1) is a sentinel node located in a minor lymphatic basin along the lymphatic drainage to a major classical nodal basin. Epitrochlear, popliteal, lower neck, triangular intermuscular space (TIS), internal mammary, paravertebral, and intraabdominal (paraaortic and retroperitoneal) may be considered uncommon sites of sentinel nodes, because these are anatomically recognized lymphatics areas for which a described surgical procedure exist. “Interval” (Figure 2) is a sentinel lymph node lying anywhere along the lymphatics between the primary tumor site and the nearest lymphatic basin, like intramuscular or subcutaneous nodes of the chest wall, deep back, flank, and occipital areas (Table 1). Interval nodes are not in an anatomical lymphatic basin and may be identified only by accurate dynamic lymphoscintigraphy. This classification reflects a different lymphatic pathway and peculiar surgical and therapeutical options.

## 3. Clinical Implication and Surgical Management

The introduction of the sentinel node procedure with dynamic lymphoscintigraphy opened a new era in the management of lymph node staging in oncology. Functional study of the lymphatic pathway has changed the approach to nodal staging at diagnosis. In the last 15 years the Sappey's rules, that governed lymphatic drainage in the last century, have been changed by dynamic lymphoscintigraphy to identify the node receiving direct lymphatic drainage from a primary tumor site. Out of classical lymph node basins, such as cervical, axillary, and groin, new “nonclassical” node sites appeared and have been reported widely in literature with different terms.

The incidence of uncommon/interval sentinel nodes varies widely in literature from 3.1% to 9.8%. This variability can be related to the different terms and definitions used to refer to an uncommon/interval node, to the different tracers used, and to the different modality of injection of the tracer.

The radiocolloids that best allow the identification of SNs are those that easily penetrate the lymphatic capillaries because of the particle sizes of 5–50 nm [1, 8]. McMasters et al., with ^99m^Tc sulfur colloid radioactive agent, reported 3.1% of interval nodes [4]. Uren et al. reported an incidence of interval nodes of 7.2% of patients using ^99m^Tc-labelled antimony trisulfide colloid that is a very small radioactive particle [3]. Roozendaal et al., like in our institution, used ^99m^Tc-labelled human albumin colloid, with an identification rate of interval nodes of 5.8% [9].

High quality lymphoscintigraphy requires specific high-resolution collimators for optimal gamma-camera imaging, with the use of detailed imaging protocols, in order to incorporate all anatomic areas to exclude uncommon/interval sentinel nodes sites. The accuracy may reflect a variable identification rate of unpredicted SNs [4, 10, 11].

Interval nodes were mostly associated with primary melanomas of the trunk. Uren among 3280 patients with cutaneous melanoma found 20% unexpected drainage from primary of the trunk. He described unexpected drainage from melanomas of the torso to the neck, to the TIS, to the subcutaneous fat over the costal margin, and also to the paravertebral, para-aortic, or retroperitoneal areas [12, 13]. The triangular intermuscular space is a well, known anatomic entity, formed by the teres major inferiorly, the infraspinatus, teres minor, and subscapularis superiorly, and the long head of the triceps laterally. Drainage to TIS will occur in approximately 25% of patients with melanoma lesions on the trunk [14].

In most cases, drainage from truncal melanomas is associated to axillary or groin drainage. Recent studies have suggested that multiple lymphatic drainage in patients with truncal melanoma, compared with drainage to just one basin, is independently associated with an increased risk of lymph node metastases and with a worse prognosis even when no pathologic lymph node involvement was identified [15, 16]. This is opposite to McHugh et al., who studied 98 out of 423 (23.2%) patients with primary truncal melanoma submitted to sentinel biopsy to multiple lymphatic basin and affirmed that multiple drainage is no an independent risk factor for nodal metastasis and has not prognostic significance. Multiple drainage must be considered only from a physiological point of view and in these cases, SN biopsy may be avoided [17].

Uncommon sentinel nodes were frequently identified for cutaneous melanomas of the extremities. In melanomas of the upper limb, Uren described drainage in the epitrochlear region in 20% of patients, in the TIS in 6%, higher than our series probably due to the different radiotracer used [12]. The incidence of popliteal SNs was related to the primary sites, as described by Thompson et al. with a rate of 6.9% [18]. From cutaneous melanoma of the head and neck area, all nonclassical sentinel nodes were identified as occipital nodes or postauricolar/mastoid nodes. These sites were considered uncommon sentinel node, because they are part of the cervical nodal chain. Lieber et al. did not detect unexpected nodes in patients with primary head and neck melanomas submitted to SN biopsy [10]. The sentinel procedure in head and neck area is often technically more difficult than other sites, because the closer of the primary tumor, and because lymph nodes are often small in size [7, 19, 20].

de Wilt et al. demonstrated sentinel nodes in discordant fields in 31.5% of patients with head and neck melanomas, but they considered postauricolar nodes, occipital nodes, preauricular nodes, cheek nodes, axillary nodes, or sentinel nodes in the TIS, as unexpected basin demonstrating that such nodes can easily be overlooked without performing high, resolution and multiple, view lymphoscintigraphy [7]. Uren reported 30% of patients with head and neck melanomas draining in unexpected basin [12].

McMasters et al. in a multicenter study and Tanabe, found the same frequency of tumor-positive sentinel nodes in unexpected and classical basin cases [4, 21]. In 11 (85%) out 13 patients with a positive unexpected sentinel node, it was the only site of disease. They concluded affirming that in cases with epitrochlear or popliteal tumor-positive sentinel node, lymphadenectomy is mandatory. For isolated interval nodes in other areas, further dissection seems to be unnecessary, out of evidence of extracapsular nodal extension.

Sumner III et al. declared that completion lymph node dissection of both the unexpected site and the regional lymph node basin upstream from it is always mandatory [22]. It is our opinion that in the absence of a parallel drainage to a classical basin revealed at lymphoscintigraphy, in the case of a positive sentinel node in an unexpected site, the elective dissection of these basins may be avoided.

## 4. Conclusions

The introduction of lymphoscintigraphy and sentinel lymph node biopsy has changed the approach to the staging of lymph node disease in oncology, permitting to identify micrometastatic nodal involvement. In rare cases dynamic lymphoscintigraphy shows sites of lymphatic drainage in nonclassical basins never described in the past when lymphatic drainage was considered only according to the anatomical proximity of the tumor primary site [23, 24]. Despite the different terms used to identify the sentinel node sites in non classical basins, the distinction between “uncommon” and “interval” sentinel node sites may classify all cases of nonclassical lymph node drainage with typical lymphatic pathway and surgical purpose.

For the uncommon sentinel node sites, like popliteal or epitrochlear, an elective dissection may be adopted, to avoid a reoperation in case of a positive sentinel node. Both for uncommon and interval tumor-positive sentinel node, the extension of surgical dissection to the nearest classical basin remains controversial but may be avoided when there is no other parallel basin of drainage identified at lymphoscintigraphy. This may be true especially for interval sites that might be considered completely ripped from the nearest classical basin. For uncommon/interval intraabdominal or intrathoracic sentinel sites, the biopsy may be avoided in favor of careful followup with clinical imaging method.

## Figures and Tables

**Table 1:** 

Uncommon SNs	Interval SNs
Epitrochlear	Chest wall
Popliteal	Deep back
Lower neck	Flank
Triangular intermuscular space	Occipital areas
Internal mammary	
Paravertebral	
Intraabdominal	
